# Restructuring of the Electricity Industry and Environmental Issues: A California Research Program

**DOI:** 10.1100/tsw.2001.71

**Published:** 2001-08-02

**Authors:** Edward L. Vine

**Affiliations:** California Institute for Energy Efficiency, Berkeley, CA, USA

**Keywords:** California, planning, aquatic resources, land use and habitat, air quality, global climate change, restructuring; public benefits, energy efficiency, research and development, environmental issues, evaluation, energy production, delivery, and use

## Abstract

As part of the restructuring of the electricity industry in many states, public benefits funding has emerged as a primary mechanism for supporting social benefits such as energy efficiency and research and development (R&D). In California, a Public Interest Energy Research (PIER) Program was established to “conduct public interest energy research that seeks to improve the quality of life for California's citizens by providing environmentally sound, safe, reliable, and affordable energy services and products. PIER includes the full range of research, development, and demonstration activities that will advance science or technology not adequately provided by competitive and regulated markets.” The PIER Program is comprised of six PIER Program funding areas, including the Energy-Related Environmental Research. The overall mission of the Energy-Related Environmental Research is to “Develop cost-effective approaches to evaluating and resolving environmental effects of energy production, delivery, and use in California, and explore how new energy applications and products can solve environmental problems.” This paper describes the process used in developing these approaches and identifies a set of environmental issues that the State plans to evaluate.

## DESCRIPTION OF THE PIER ENVIRONMENTAL AREA

In 1996, California adopted legislation that drastically revamped how California's investor-owned electric services industry operates (1996 Statutes, Chapter 854, hereinafter "AB 1890"). The legislation also authorized collection of a surcharge on retail electricity sales of not less than $62.5 million annually for four years, to ensure a continuation of public interest energy research (PIER), development, and demonstration (RD&D) projects. Because of the source of funding, the PIER Program is specifically mandated to produce public interest benefits for electricity ratepayers in California. The PIER program was established at the California Energy Commission (the Energy Commission) to implement the RD&D provision of AB 1890, funded at $61.8 million annually from January 1, 1998 to December 31, 2001. Senate Bill 90 further defined the PIER program in October 1997, identifying key program areas and administrative and funding criteria. In September 2000, the governor signed legislation (AB 995) that continues PIER program funding for another ten years (through January 1, 2012).

The Energy Commission has established six major subject areas for the PIER Program. These subject areas are: Residential and Commercial Buildings End-Use Energy Efficiency; Industrial/Agricultural/Water End-Use Energy Efficiency (Process Energy); Renewable Energy Technologies; Environmentally Preferred Advanced Generation; Strategic Energy Research; and Energy-Related Environmental Research.[*]

As one of the six major subject areas, Energy-Related Environmental Research (otherwise called the *PIER Environmental Area*, or *PIEREA*) is responsible for addressing the environmental impacts and uses of electricity in California. As defined by the PIER strategic plan, the overall mission of the PIEREA is to:

> "Develop cost-effective approaches to evaluating and resolving environmental effects of energy production, delivery, and use in California, and explore how new energy applications and products can solve environmental problems."

Protecting and improving the environment is a major element of planning in each of the six subject areas. The mission of PIER is to conduct energy research to improve quality of life by "…providing *environmentally sound*, safe, reliable and affordable energy services and products…." The research conducted in PIEREA is therefore crosscutting; the research will cut across several subject areas and disciplines. In addition to addressing suspected and documented environmental impacts of electricity, PIEREA provides basic scientific information and tools for understanding the environmental implications of related technology and fuels choices that may be undertaken elsewhere in the PIER Program.

## PLANNING PROCESS

PIEREA recently established a long-term (at least five-year) research plan that targets energy-related environmental research projects for PIER program funding in four subject areas (California Energy Commission 2001):

1. Aquatic resources
2. Land use and habitat
3. Outdoor air quality
4. Global climate change

[*] More information about the mission, goals and objectives, and funded research of the PIER Program can be found at www.energy.ca.gov/research/PIER/index.html.

The major goal of this effort is to identify, develop, evaluate, refine, and select RD&D initiatives that address major energy-related environmental issues for California to be funded through PIEREA. This plan provides a broad framework and justification for selected RD&D initiatives based on: (1) planning criteria used to assess the relative importance of environmental issues; (2) an RD&D program planning-level methodology for assessing benefits, costs, and other impacts across subject areas as part of a process of developing an integrated plan; and (3) an overall RD&D program planning rationale for discriminating between potential high-priority and low-priority RD&D initiatives. The plan addresses ways of improving scientific understanding of the adverse public health and environmental impacts attributable to the generation, transmission and distribution, and use of electricity, as well as ways of developing mitigation/enhancement strategies, tools, or technologies to address these adverse impacts.

The long-term PIEREA Environmental Planning Process is outlined in Fig. 1. To develop this plan, the PIEREA team collected and analyzed the following types of information:

1. The major trends and drivers affecting electricity and the environment in California
2. Key current and future issues and rationales for their possible inclusion in PIEREA
3. Existing environmental research efforts relating to key electricity-related issues identified, conducted by the Commission and other organizations
4. Major gaps in environmental research relating to issues

Through this process, 29 environmental issues were first identified. Using a list of evaluation criteria, the team developed a shorter list of high priority environmental issues for near-term attention. The team used a modified Delphi method for analyzing the issues and making issue selections for consideration by the Commission, i.e., the issues were evaluated in one round by the team and, after discussion, the issues were re-evaluated a second time by the same team members. The evaluation criteria formed the basis for discussions and ultimate selections by the team. For the eleven high priority issues identified, more specific research plans or roadmaps will be developed by planning teams with specific expertise relative to the selected issues that include long-, mid-, and short-term goals, milestones, and strategies for addressing the issues.

The plan is intended to be a living document and will be updated to remain current with end-user needs, with the state of the science in the subject areas being addressed, and with pending legislative or regulatory decisions. The plan is a portfolio of environmental research issues of both short-term and long-term relevance. Similarly, research activities addressing these issues will include a mix of efforts having short-term and long-term duration.

The PIEREA team sought the advice of several stakeholders on the merits of the identified issues. The stakeholders represented key state and federal environmental regulatory agencies (e.g., U.S. Environmental Protection Agency and California Department of Fish and Game), utilities (e.g., Southern California Edison Company), environmental non-governmental organizations (e.g., Environmental Defense), and industry groups (e.g., California Manufacturers and Technologies Association and the Geothermal Energy Association). Throughout the plan’s implementation, stakeholder involvement will remain crucial to ensure that the proposed research is relevant and that results are communicated effectively. Stakeholder participation will increase the credibility of the planning effort and build public support. In addition, inclusion of stakeholders in the planning process promotes understanding of and interest in the findings resulting from the planning process.

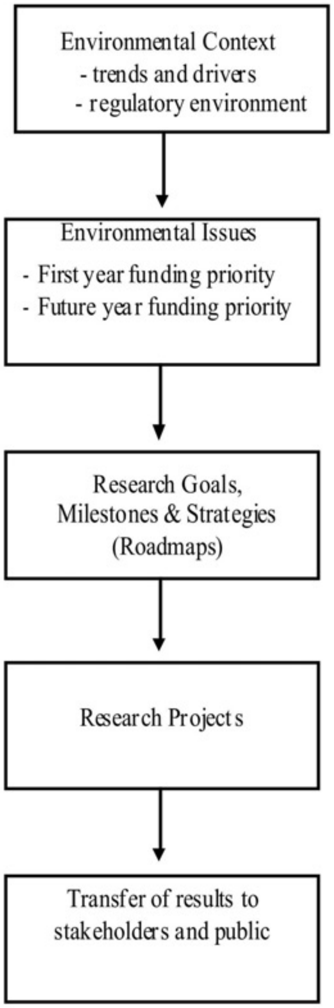

**FIGURE 1.** PIEREA environmental planning process.

## CROSSCUTTING DRIVERS AND TRENDS*

The generation, transmission and distribution, and use of electricity in the State are affected by market, technological, demographic, and regulatory *drivers* and *trends*. Each of these drivers and trends affects the State's environment and the State's energy system and determines which environmental issues the State must address. Due to space constraints, we briefly describe the key crosscutting drivers and trends that affect these subject areas and then indicate graphically how the key drivers and trends lead to particular environmental concerns and issues (Figs. 2–5).

Many crosscutting drivers and trends affect the generation, distribution, and use of electricity in California. As a result of these drivers and trends, new generation facilities will need to be built in the near future to meet an increased energy demand, and these facilities will need to be licensed and sited. The following subsections discuss the crosscutting drivers and trends that affect the development of new California power plants.

* There were other trends and drivers that were unique to each subject area; they are not discussed in this section because, unlike crosscutting trends and drivers, they did not affect more than one subject area.

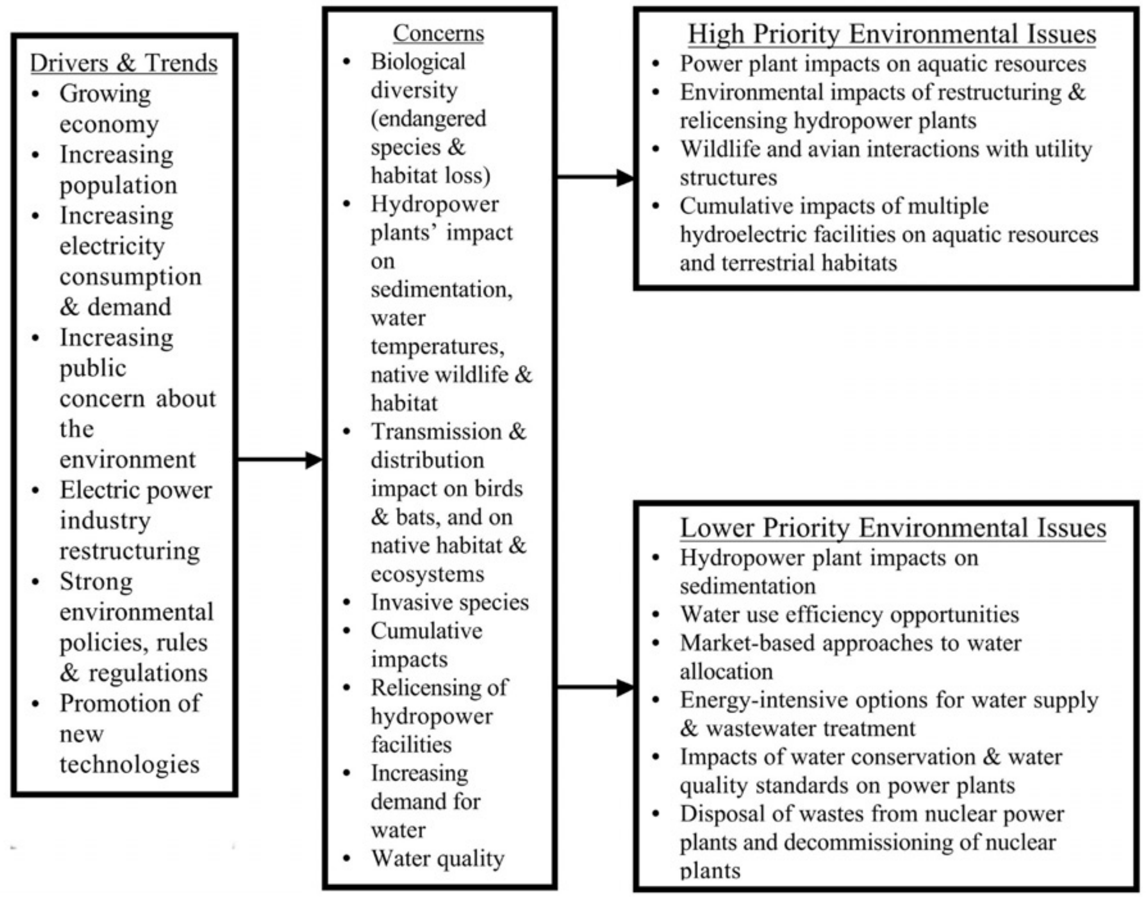

**FIGURE 2.** Aquatic resources and land-use habitat.

## Growing Economy

California's economy has picked up momentum in recent years (especially in Northern California), led by such diverse sectors as international trade, movie production, tourism, and high-technology manufacturing and services. This growth has led to an increased demand for energy in the residential, commercial, industrial, and agricultural sectors, which will require new generation facilities.

## Increasing Population

California's population is expected to reach approximately 50 million people in the next 20 years, and much of this growth is expected to occur in the State's hot inland valleys. This growth will lead to more housing and greater energy and related infrastructure demands in providing the services needed for a growing population.

## Increasing Electricity Consumption and Demand

With the predicted future increases in economic and population growth, electricity consumption is expected to grow at a rate of approximately 2%/year for the 2000–2010 timeframe. The statewide peak demand is expected to grow at a rate of 1.7%/year during that same period. New generation facilities will be needed to meet this energy demand.

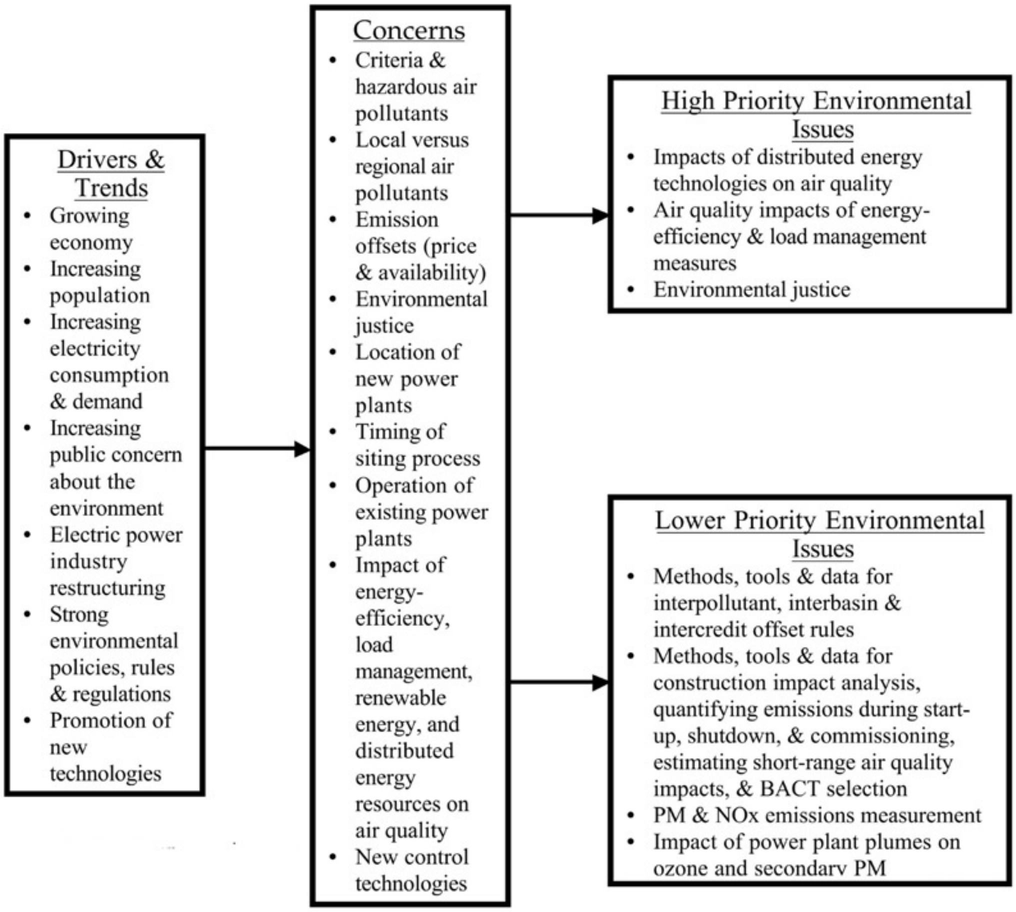

**FIGURE 3.** Outdoor air quality.

## Increasing Public Concern about the Environment

The California public is very sensitive to environmental and public health issues, including deteriorating air and water quality, unsafe conditions, loud noise, visual blight, electromagnetic radiation, habitat loss and degradation, decreasing biodiversity, toxic contamination of soil and water, and global warming. The public's concern for clean air and water will continue to affect the future of energy use in the state, e.g., favoring the introduction of clean energy technologies such as energy efficiency and renewable energy technologies.

## Electric Power Industry Restructuring

Although California's restructuring legislation (AB 1890) preserves California's commitment to developing diverse, environmentally sensitive electricity resources, the net effects of restructuring depend on many factors: underlying fuel markets, existing capacity mix, the type of regulatory changes accompanying restructuring (e.g., a renewable portfolio standard, production tax incentives for renewables, a public benefit charge, and removal of regulatory barriers to combined heat and power and distributed power), and demand responses to price changes. The mechanics of restructuring and the potential changes in environmental regulations and effects are uncertain, requiring ongoing research.

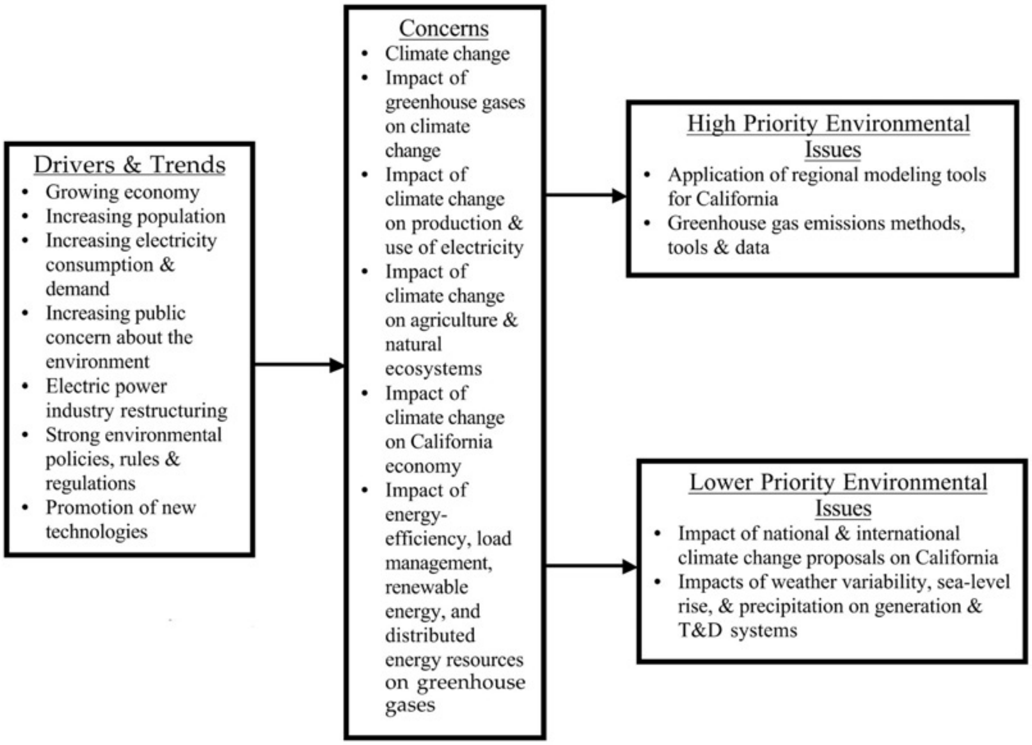

**FIGURE 4.** Global climate change.

Since the summer of 2000, California utilities have experienced high prices for electricity on the wholesale market and supply problems that caused electricity outages and rolling blackouts in California. In August 2000, the California Legislature passed a number of bills to address these electricity system issues. For example, AB 970 recognizes that, as a result of the increased demand for electricity, a serious lag in the construction and operation of new generation facilities, and insufficient energy-efficiency measures, California may face serious electricity shortages over the next two years. Under AB 970, the Energy Commission is able to certify certain electrical generation facilities through an expedited review and approval process if they satisfy specific conditions that ensure that the facility will not pose significant adverse effects on the environment as a result of construction or operation. However, measures such as this may be insufficient for providing the electricity necessary to supply California's needs, and a continued electricity shortfall has created pressure to relax environmental standards as a means to increase the supply of electricity.

## Strong Environmental Policies, Rules, and Regulations

California has an environmental regulatory infrastructure (consisting of local, regional, state, and federal agencies) that has promulgated environmental policies, rules, and regulations that address many of the environmental issues confronting California. These rules and regulations are intended to ensure that issues are identified and appropriate environmental mitigation measures are applied to protect the health and welfare of the citizens of California. If environmental impacts deemed significant cannot be avoided, mitigation measures must be identified and implemented to offset the impact and contribute to recovery. Although they are recognized as among the most stringent in the nation, there is broad consensus that these rules and regulations are not a major contributor to the current energy crisis in California.

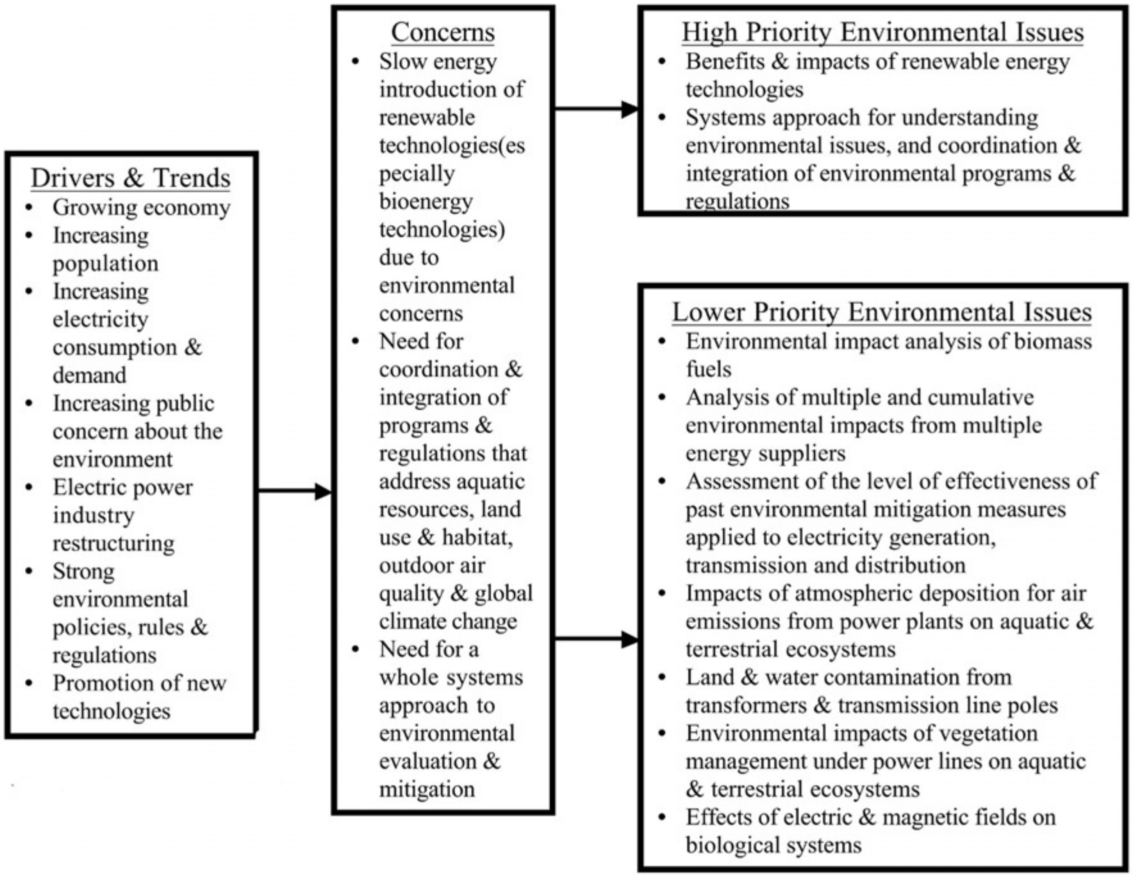

**FIGURE 5.** Crosscutting issues.

## Promotion of New Technologies

Technology development and dissemination is a dynamic field that affects both the supply and use of energy. Recently, the central generation station technology of choice has been natural gas–fueled combined-cycle turbines. Use of advanced exhaust gas cleanup and cleaner, more efficient systems has led to significant, steady emission reductions per unit of electricity generated when compared to older fossil-fired boilers. Renewable energy technologies and, in particular, distributed energy resources (DER) such as solar cells, microturbines, and fuel cells are being promoted by state and federal governments. On the demand side, the introduction and commercialization of new energy-efficiency technologies and services (including some electrotechnologies) are improving the energy efficiency of the residential, commercial, industrial, and agricultural sectors. Significant new development of wind, geothermal, and small hydroelectrical generation sources is also likely in the coming decade. Other technologies (e.g., fuel cells) are still in the early stages of commercialization.

## HIGH PRIORITY ENVIRONMENTAL ISSUES

A group of environmental issues was selected that would be the focus for research funding for the coming year. The selection of these "high-priority" environmental issues was based on an analysis of drivers and trends and, most importantly, on an evaluation process that systematically examined a proposed list of 29 environmental issues. The proposed list was an outcome of the analysis of drivers and trends described in the previous section, which was based on a review of the literature and interviews with key stakeholders. A set of 23 criteria were used to evaluate the proposed list of environmental issues. These criteria included the following: perceived urgency; statewide significance; degree to which the issue is being addressed by others; probability for developing innovative solutions; and the potential for cost sharing. The evaluation criteria and measurement scale were used to help the reviewer better understand the issue and ultimately determine if it warranted first-year funding, a scoping study (smaller-scale investigative study of the issue), or consideration at a later date. For the measurement scale, higher numbers did not necessarily represent greater importance or value and were not counted as such; however, they helped the reviewer to understand the scope and the relative urgency of the issue. The evaluation process resulted in the selection of the following 11 environmental issues.

## Aquatic Resources and Issues

***Power plant impacts on aquatic resources.*** Electric power plants that use water for power production or cooling alter or eliminate natural ecological and hydrological functions in aquatic systems. These plants affect riverine, estuarine, and marine systems, which have significantly contributed to aquatic species decline. Adverse impacts include fatality from impingement (i.e., trapping aquatic organisms in intake screens) and entrainment (i.e., passing aquatic organisms through cooling systems and pumping intake valves and turbines); blockage of fish movement and migration; fragmentation of ecosystems; and alterations in normal stream flows and temperatures. Hydroelectric plants that use water for energy production can impact aquatic resources through alteration of upstream and downstream habitat and by entrainment and impingement.

***Environmental impacts of restructuring and relicensing of hydropower plants.*** Both electric power industry restructuring and the relicensing of hydropower projects are expected to affect the environmental management and stewardship of land and water resources by owners, including the potential for changes in peak power production and a shift in resource priorities. There is a need to better identify and understand these impacts.

***Cumulative impacts of multiple hydroelectric facilities on aquatic resources, as well as terrestrial habitats.*** The cumulative impacts of multiple hydroelectric facilities on aquatic resources and terrestrial habitats in a watershed are difficult to evaluate, due to a lack of site-specific information and appropriate methodologies.

## Land Use and Habitat Issues

***Wildlife and avian interactions.*** Wildlife and avian interactions with utility structures can result in electrocutions on poles used for distribution lines and collisions with transmission line conductors or wind turbines and supporting guy wires. Such interactions can result in negative impacts to birds, costly power outages, and violations of State and federal laws. Transmission line systems can cumulatively contribute to habitat loss and degradation, the primary factors leading to species endangerment and decreased biodiversity.

## Outdoor Air Quality Issues

***Impacts of distributed energy technologies on air quality.*** There is a need for improved methods, tools, and data to estimate impacts of emerging energy technologies (e.g., distributed energy) and fuels on air quality.

***Air quality impacts of energy-efficiency and load management measures.*** There is a need for improved methods, tools, and data to quantify the air quality impacts of energy efficiency and load management measures for preparing air quality management plan baselines and as offsets or emission reduction credits.

***Environmental justice.*** Electricity generators and the development of transmission and distribution infrastructure can increase local air emission impacts and place a disproportionate burden of those impacts on local minority and low-income communities.

## Global Climate Change Issues

***Application of global circulation modeling results to California.*** There is a need for improved methods and tools to translate global circulation modeling results to California regional climate, so that researchers can analyze the impacts of global climate change in California and an evolving electricity system in particular.

***Greenhouse gas emissions methods, tools, and data.*** There is a need for improved methods, tools, and data to (1) develop simple and accurate guidelines to estimate the greenhouse gas (GHG) emissions reductions in power plants that are attributable to the implementation of electricity conservation efforts; (2) prepare comprehensive inventories of GHG emissions (e.g., $CO_2$ emissions and their sources, methane emissions from the operation of hydropower facilities and other sources, $N_2O$ emissions and their sources, and other GHG emissions and their sources); and (3) develop supply curves of GHG emissions reduction options.

## Crosscutting Issues

***Coordination and integration of environmental programs and regulations.*** When addressing the environmental impacts related to the generation, distribution, and use of electricity, concerns about aquatic resources, land use and habitat, air quality, and global climate change are intimately related. A whole systems approach is needed for understanding the interactions of all the parts of the system, including growth, economic development, and new technologies; the influence of regulatory requirements; and how the impacts, benefits, and tradeoffs of different scenarios affect energy development and impact the environment. Therefore, there is a need to coordinate and integrate programs and regulations that address aquatic resources, land use and habitat, air quality, and global climate change to avoid future penalties to the State economy from costly uncoordinated efforts.† [*]

***Benefits and impacts of renewable energy technologies.*** There is a need for improved methods, tools, and data to estimate the benefits and impacts of emerging technologies (e.g., renewable energy).†

[*] Nine environmental issues are areas targeted for funding full-scale research projects. The two crosscutting issues (denoted with a dagger (†)) require preliminary scoping studies to determine whether full-scale research projects should be initiated.

## CONCLUSIONS

### Opportunities for Evaluators

As one can imagine, there are many opportunities for evaluators in analyzing environmental issues related to restructuring. While each state in the U.S. has its own unique inventory of energy sources and emissions, many states will be confronted with similar drivers and trends affecting California — even without the restructuring of the energy industry. Example questions are provided below to illustrate the type of evaluation that is needed:

- How effective has been the redesign of transmission and distribution towers for mitigating avian and bat fatalities?
- What are the best strategies for removing sediment behind dams, and is there a market for the sediment?
- Which types of distributed energy systems will improve air quality and how does this affect the entire energy system?
- How will climate change affect temperature and precipitation patterns and their impact on electricity consumption?
- What are the costs and benefits of bioenergy technologies?

Many of these questions raise other questions and will challenge the evaluation community to evaluate issues at a larger scale and at a more complex level. A systems approach will be needed for making sure all the key interactions and feedback loops are carefully examined, e.g., examining the impacts of a biomass energy project on land use, aquatic systems, and air quality. Finally, as envisioned in California's case, both short- and long-term research will be needed; evaluators must look beyond today's pressing energy needs to a more distant future with possibly different energy systems (e.g., those primarily based on energy efficiency and renewable energy technologies).

### Next Steps

For the eleven high priority issues identified in the plan, more specific research plans or roadmaps are being developed by planning teams with specific expertise on the selected issues that include long-, mid-, and short-term goals, milestones, and strategies for addressing the issues. After these plans have been completed, the final step is the implementation of the respective RD&D plans. This step will require coordination with other regulatory agencies, formation of project steering committees, solicitation of proposals, executing contracts with researchers, and transferring research results to stakeholders.

### Final Thoughts

In designing energy policies and promoting technologies in a more competitive environment, each State undergoing restructuring will need to understand the environmental implications of such policies and technologies. States pursuing the restructuring of the energy industry have an obligation to consider the environmental benefits and costs of energy policies. By developing public interest energy research and development programs, some states are preparing themselves for addressing these issues during restructuring. At the same time, there is a significant opportunity to coordinate and integrate energy and environmental policies at the state level by paying more attention to the interrelated aspects of outdoor air quality, water resources, land use and habitat, and global climate change. Evaluators will be playing a critical role on addressing these issues.

## ACKNOWLEDGMENTS

I would like to thank the following people for their assistance in preparing the Energy Commission report and for participating in the planning process described in this paper: G. Barkalow, K. Birkinshaw, J. Cole, G. Franco, M. Layton, J. Lents, J. O'Hagan, S. Peck, J. Quinn, J. Sharpless, L. Spiegel, and M. Wilson. I would like to acknowledge key funding for this work from the California Institute for Energy Efficiency (CIEE), a research unit of the University of California. Publication of these findings does not imply CIEE endorsement of or agreement with these findings, nor that of any of their sponsors.
